# A Green Fluorescent Protein with Photoswitchable Emission from the Deep Sea

**DOI:** 10.1371/journal.pone.0003766

**Published:** 2008-11-19

**Authors:** Alexander Vogt, Cecilia D'Angelo, Franz Oswald, Andrea Denzel, Charles H. Mazel, Mikhail V. Matz, Sergey Ivanchenko, G. Ulrich Nienhaus, Jörg Wiedenmann

**Affiliations:** 1 Institute of General Zoology and Endocrinology, University of Ulm, Ulm, Germany; 2 Department of Internal Medicine I, University of Ulm, Ulm, Germany; 3 NightSea, Andover, Massachusetts, United States of America; 4 Integrative Biology, University of Texas in Austin, Austin, Texas, United States of America; 5 Institute of Biophysics, University of Ulm, Ulm, Germany; 6 Department of Physics, University of Illinois at Urbana-Champaign, Urbana, Illinois, United States of America; 7 National Oceanography Centre, University of Southampton, Southampton, United Kingdom; The Beatson Institute, United Kingdom

## Abstract

A colorful variety of fluorescent proteins (FPs) from marine invertebrates are utilized as genetically encoded markers for live cell imaging. The increased demand for advanced imaging techniques drives a continuous search for FPs with new and improved properties. Many useful FPs have been isolated from species adapted to sun-flooded habitats such as tropical coral reefs. It has yet remained unknown if species expressing green fluorescent protein (GFP)-like proteins also exist in the darkness of the deep sea. Using a submarine-based and -operated fluorescence detection system in the Gulf of Mexico, we discovered ceriantharians emitting bright green fluorescence in depths between 500 and 600 m and identified a GFP, named cerFP505, with bright fluorescence emission peaking at 505 nm. Spectroscopic studies showed that ∼15% of the protein bulk feature reversible ON/OFF photoswitching that can be induced by alternating irradiation with blue und near-UV light. Despite being derived from an animal adapted to essentially complete darkness and low temperatures, cerFP505 maturation in living mammalian cells at 37°C, its brightness and photostability are comparable to those of EGFP and cmFP512 from shallow water species. Therefore, our findings disclose the deep sea as a potential source of GFP-like molecular marker proteins.

## Introduction

In recent years, GFP-like proteins from marine invertebrates have emerged as indispensable tools for tracking of proteins or cells, sensing of intracellular conditions such as Ca^2+^ or pH variations and for studies of protein interactions and gene activity, opening several stimulating perspectives in live cell imaging [1]–[9]. The success of these pigments as molecular markers results mainly from their unique capability to form a chromophore autocatalytically; no additional cofactors are required, only molecular oxygen [10], [11]. Recently, the group of photoactivatable GFP-like proteins attracted particular attention. The fluorescence of these proteins can either be switched on or off (paGFP, KFP1, Dronpa, mTFP0.7) or the emission color can be shifted towards longer wavelengths (Kaede, KikGR, EosFP, Dendra, mcavRFP, cjarRFP, lhemOFP, PS-CFP) by irradiation with light of specific wavelengths [3], [12]–[21]. The possibility to control the fluorescence properties enabled numerous innovative applications, for instance, regional optical marking and tracking of cells, subcellular organelles or proteins [3], [15], [21]. Additionally, photoswitchable or photoconvertible proteins can be employed in novel microscopy concepts that allow imaging of subcellular structures at a resolution beyond the diffraction barrier of optical microscopy [22]–[24]. The swift evolution of super-resolution microscopy techniques calls for novel and optimized photoswitchable FPs [6], [23], [25].

The application of FPs derived from marine species has been further extended by protein engineering. Optimized variants were created that feature faster and more complete maturation of the chromophore, shifted excitation and emission wavelengths, reduced aggregation and oligomerization, brighter fluorescence and increased photostability [15], [17], [26]–[34]. However, the development of marker proteins with entirely novel spectral properties greatly relies on the discovery of natural lead structures. To date, GFP-like proteins have been detected in different taxa of marine invertebrates that inhabit the photic zone of the ocean [35], [36]. Most members of this protein family were isolated from cnidarians, mainly from the taxon anthozoa, but GFPs were also found in copepods (crustacea) and amphioxus (chordata) [2], [7], [21], [36]–[44].

The expression of GFP-like proteins in marine invertebrates has been linked with the photophysiology of the animals in various ways. Potential functions of the pigments include photoprotection, optimization of symbionts photosynthesis, photoreception, or inter- and intra- species signaling [36], [38], [45]–[49]. In a large number of scleractinian corals, the expression of GFP-like proteins is tightly regulated on the transcriptional level by the intensity of blue light [50]. However, color morphs of the Mediterranean sea anemone *Anemonia spp.* and the Caribbean coral *Montastrea cavernosa* are defined by different expression levels of distinct sets of GFP-like proteins that appear to be fixed rather than regulated by light [18], [51]–[53]. Moreover, the recent discovery of FPs in non-zooxanthellate anthozoans suggests putative functions of these pigments that are independent of the light requirements from symbiotic associations with zooxanthellae [37], [43], [48]. The possibility of light-independent expression of FPs encouraged us to search the dark depths of the ocean as a potential new source of GFP-like proteins.

## Results and Discussion

### Identification of a GFP-like protein in a deep sea ceriantharian

**Figure 1:**
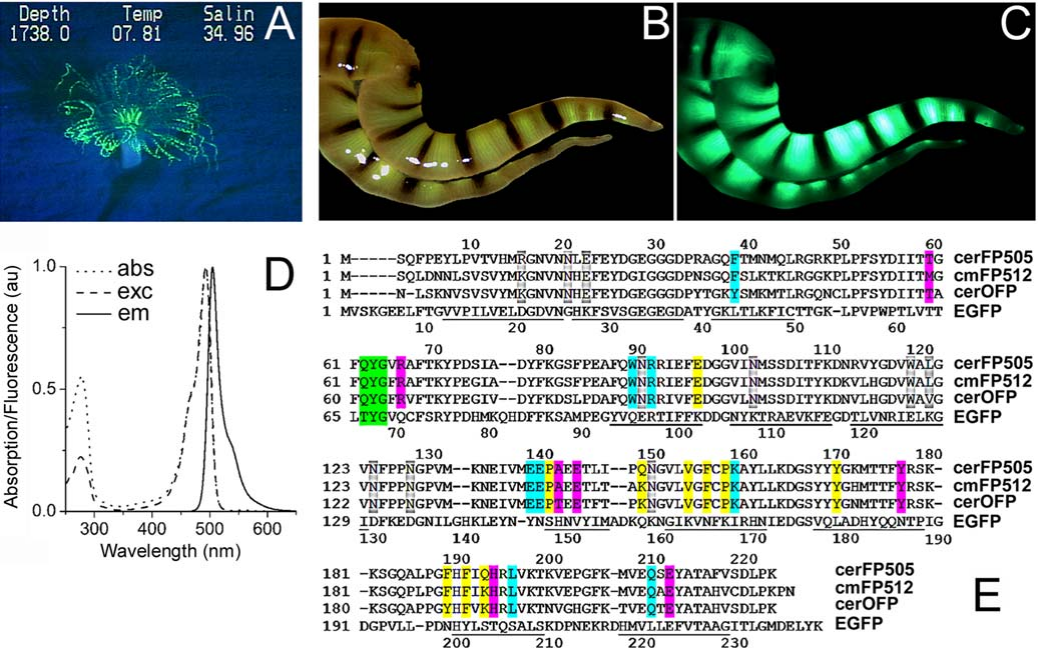
Identification of a GFP-like protein from a deep sea ceriantharian. A) Fluorescence image of a ceriantharian acquired in 530 m depth in the Gulf of Mexico. The depth (ft), temperature (Temp, °C) and the salinity (Salin) measured during image acquisition are displayed in the upper part of the panel. B–C) Microscopic image of two tentacles photographed under white light (B) or blue light excitation using a GFP-filter set (C). D) Absorption, excitation and emission spectra of purified recombinant cerFP505. Fluorescence spectra were recorded using 450 nm light for excitation, whereas the emission was collected at 550 nm. E) Multiple alignment of amino acid sequences from ceriantharian fluorescent proteins and EGFP. The chromophore – forming tripeptide is highlighted in green. Residues interacting directly with, or found in the proximity of, the chromophore in cmFP512 are labeled in blue or red, respectively. Amino acids underlayed in grey or yellow are involved in A/B or A/C interface interactions in cmFP512, respectively [59]. The numberings for cerFP505 and EGFP are given above and below the sequences, respectively. Regions forming ß-sheets in EGFP are underlined.

As part of the project “Operation Deep Scope” (NOAA Ocean Explorer), we screened benthic habitats of the Gulf of Mexico for fluorescent organisms. For this purpose, the *Johnson-Sea-Link II* submersible (Harbor Branch) was equipped with four lights, two of which (400 W HMI lights, Deepsea Power & Light, San Diego, USA) were mounted on the front edge of the work basket so that they could be positioned close to the sea floor. They were fitted with custom blue short-pass (cutoff at approximately 460 nm) interference filters (NightSea, Andover, MA) mounted in custom filter holders that minimized stray light while allowing water flow for cooling the lamp. The science observer in the sphere wore barrier filter glasses (NightSea, Andover, MA) that blocked the reflected blue light so that any fluorescence could be seen with high contrast, and the observer would call the pilot's attention to features of interest on the sea floor. A fluorescence barrier filter (long-pass acrylic absorption filter, 50% transmission at approximately 500 nm) (NightSea, Andover, MA) was positioned in front of the submersible's video camera using a hinged mount, so that the pilot could use the submersible's manipulator arm to pivot the filter away from the camera to acquire conventional white light images to contrast with the fluorescence images collected with the filter in place. The special detection equipment allowed to document the fluorescence of several fluorescent deep sea organisms. Specimens showing particularly striking green fluorescence were found among the taxon ceriantharia (Figure 1A). The presence of this taxon in the West Atlantic had previously been reported off the coast of New England down to 1600 m depth [54]. To date, two species (*Ceriantheopsis americanus*, *Cerianthus borealis*) are known from Nova Scotia to Cape Hatteras [54], and three species (*Pachycerianthus curacaoensis*, *Ceriantheopsis americanus*, *Isarachnanthus maderensis*) from the Caribbean and the Gulf of Mexico, all from shallow water down to a depth of 150 m [55]–[57]. We collected tentacle samples from an individual displaying green fluorescence found in 530 m depth at the “Green Canyon” site. Using white light illumination, the view of the tentacles under the stereomicroscope is dominated by a pattern of dark pigment rings (Figure 1B). The lighter regions show a brownish color with a yellow-green tint. These regions emit intense green fluorescence peaking at ∼504 nm, if excited with blue light (Figure 1C). In contrast, the dark rings are non-fluorescent. The tentacles were preserved in RNA*later* for subsequent analysis. Examination of the cnidom of marginal tentacles revealed the absence of p-mastigophores, thus the animal can be considered to belong to the taxon Spirularia [58]. Spirularia encompasses two families, Botrucnidiferidae and Cerianthidae. The cnidoms of known members of these two families from the region could not be matched with the deep-sea ceriantharian of the present study. Therefore, the specimen might represent a new species (T. Molodtsova, personal communication). Future work should clarify the taxonomic status of the green fluorescent deep sea ceriantharian.

We isolated total RNA from the preserved tentacle tissue and synthesized cDNA. Fragments coding for a GFP-like protein were amplified by PCR using internal primers designed to be complementary to the sequence of cmFP512 in combination with cDNA adapter primers. Subsequently, primers specific for the 5′ and 3′ termini of the open reading frames were synthesized and the full length cDNA was amplified, cloned and sequenced. The coding sequence was deposited at GenBank under the accession number EU927368. The amino acid sequence displays 83% sequence identity with cmFP512 and 78% with an orange fluorescent protein from *Cerianthus sp.* (OFP) (Figure 1E). Like cmFP512 and OFP, the GFP-like protein from the deep sea ceriantharian shows a remarkable difference from other representatives of the protein family, for instance, it shares only about 40% identical amino acids with anthozoan FPs such as Kaede, EosFP, eqFP611 and Dronpa [12], [15], [21], [44]. The protein was expressed in *E. coli* and a GFP was purified that had excitation and emission maxima at 494 and 505 nm, respectively (Figure 1D). Therefore, the GFP was named cerFP505 (ceriantharian fluorescent protein with an emission maximum at 505 nm). The elution profile of cerFP505 obtained from size exclusion chromatography evinced a tetrameric structure typical for many anthozoan GFP-like proteins including cmFP512 [43], [59]–[62]. Analysis by SDS-PAGE showed that the subunits form an unfragmented, single band associated with a molecular mass of ∼26 kDa (data not shown).

The chromophore in cerFP505 is formed by the tripeptide Gln^62^-Tyr^63^-Gly^64^ (Figure 1E). As obvious from the excitation maximum at 494 nm, the side chain of Tyr^63^ exists predominately in the deprotonated state. In contrast, GFP from *Aequorea victoria* required the Ser^65^Thr mutation to transfer the neutral chromophore to the deprotonated state. Glutamine in the first position of the chromophore is common for both green and red fluorescent proteins from anthozoans such cmFP512, asFP499, OFP from *Cerianthus sp.* or dsRed [41], [59], [63]. All residues homologous to amino acids interacting with the chromophore in cmFP512 are conserved in cerFP505 [59]. Only one amino acid of cmFP512 with a side chain localized in the proximity of the chromophore, Met59, is exchanged by Thr in cerFP505. This alteration seems to be quite common among anthozoan FPs, since threonine is also found in the corresponding position in OFP from *Cerianthus sp.*, EosFP and Dronpa [15], [21], [63]. Further differences between cerFP505 and cmFP512 are three conservative substitutions of residues involved in the formation of the A/B (Leu15Arg) and A/C interfaces (Lys149Gln and Lys193Gln) within the tetrameric assembly. Interestingly, the emission maximum of OFP from *Cerianthus sp.* is shifted to 573 nm, although the chromophore and its environment appear to be highly conserved in comparison to ceriantharian GFPs. A potential key residue that might be responsible for the red shift of OFP is Thr142. In cmFP512 and in cerFP505, this position is occupied by Val. In contrast to Ser142 in other FPs, this residue is not directly involved in positioning the phenolate group of the chromophore in cmFP512 [60], [62], [64]. Instead, the sidechain of Tyr63 interacts with Lys159 and surrounding water molecules [59]. The introduction of the hydroxyl group of Thr142 will most likely interfere with this hydrogen bonding network and result in a repositioning of the phenolate of the chromophore. The slightly altered conformation of the chromophore might facilitate reactions that extend the conjugated π-sytem, for instance, by formation of a third heterocycle as in the dsRed derivative mOrange [33], [65].

### Reversible photoswitching properties of cerFP505

In the spectral range beyond 400 nm, the absorption spectrum of the native protein overlapped completely with the excitation spectrum, while that of the alkali-denatured protein (measured at pH 13) displayed a maximum at 448 nm, typical of the GFP-type chromophore [66], [67]. An extinction coefficient of ε_494 nm_ = 54000 M^−1^ cm^−1^ and a fluorescence quantum yield of 0.55 was calculated for cerFP505. These values are comparable to those obtained for cmFP512 and place the pigment in the group of highly fluorescent proteins (Table 1) [43].

**Table 1:** Photophysical properties of ceriantharian fluorescent proteins.

Protein	Excitation/Emision maxima (nm)	Stokes shift (nm)	Fluorescence quantum yield	Extinction coefficient (M^−1^ cm^−1^)	Bleaching half-life (min)^a^
cerFP505	494/505	11	0.55	54000_(on)_	7±1.0
				∼46000_(off)_	
cmFP512	503/512^a^	9	0.66^a^	58,800^a^	12±2.0
OFP	548/573^b^	25	0.64^b^	60,000^b^	–
EGFP	489/509^c^	20	0.60^c^	53,000^c^	13±0.7

a values calculated from the data in Figure 3C.

b data from [43].

c data from [63].

d data from [81].

**Figure 2:**
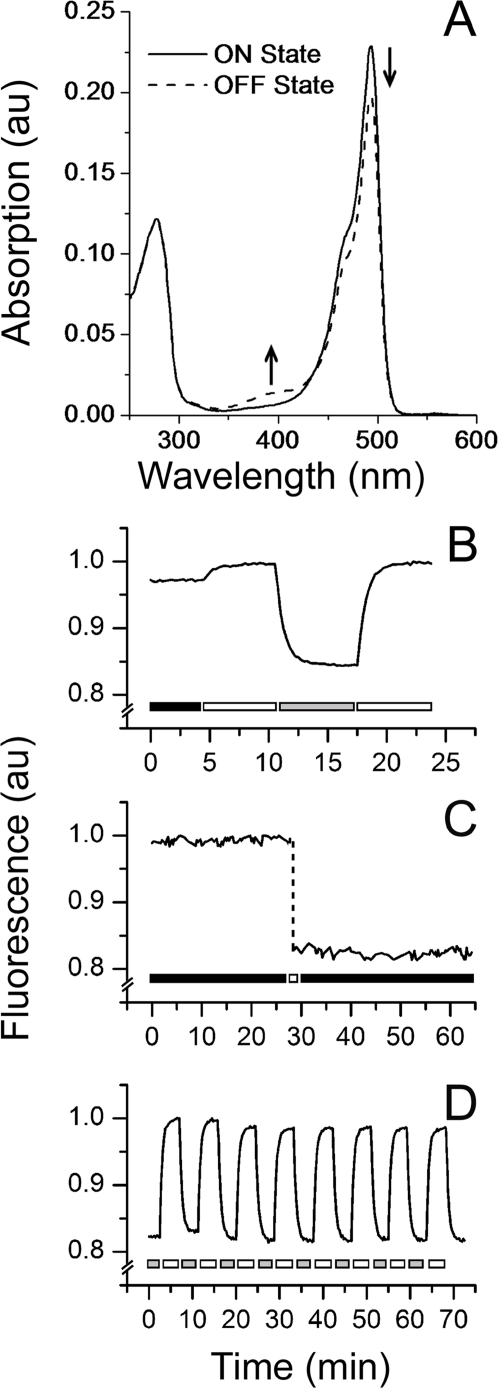
Photoswitching of the chromophore in cerFP505. A) Absorption spectra after irradiation with 400 nm (on-state) or with 450 nm light (off-state). Deactivation gives rise to a second peak around 390 nm. B) Ground state equilibrium. Irradiation at 400 nm drives the protein to the on-state, 450 nm light turns off the “activatable” fraction of the protein. Bars above the x-axis specify the light treatment, 400 nm light (white bar), 450 nm light (grey bar) or no light (black bar). C) Fluorescence emission of the protein in the on- or off-state was recorded for ≥30 min in the dark after 5 min irradiation with 400 or 450 nm light. Bars above the x-axis specify the light treatment as described above. D) The reversibility of the reaction is shown for eight cycles of activation/deactivation. Bars above the x-axis specify the light treatment as described in (B).

While studying the photobleaching behavior of cerFP505, we found that blue-light irradiation (∼450 nm) resulted in a fast initial decrease of the green fluorescence emission. Full fluorescence intensity could be recovered by illumination of the sample with light of ∼400 nm. To analyze this effect in detail, absorption spectra of purified protein samples were collected after 5 min illumination with 400 nm (on-state) or with 450 nm (off-state) light (Figure 2A). Both spectra displayed maxima at 494 nm, however the amplitude of the peak was ∼15% higher for the sample in the on-state. The deactivation of the chromophore was reflected in the lower extinction coefficient (ε_494 nm_≈46,000 M^−1^ cm^−1^) calculated for the protein in the off-state at 494 nm, whereas the quantum yield remained unaltered. A second peak at ∼390 nm appeared at the expense of the decreasing 494 nm absorption band. As the absorption at 390 nm is characteristic for the neutral GFP-type chromophore [68], this finding suggests that a reversible protonation of the chromophore is involved in the photoswitching process [68]. Reversible protonation and *cis* – *trans* isomerization of the chromophore were recently found to be responsible for the on/off switching of the GFPs Dronpa and mTeal0.7 [13], [15], [69], [70]. However, in contrast to Dronpa and mTeal0.7 that can be switched off essentially completely, the cerFP505 fluorescence intensity can be reduced only by ∼15%. This percentage shows slight variations among different protein preparations, but is stable within a defined sample.

In order to determine the ground state equilibrium of the fluorescent and the non-fluorescent states of cerFP505, the protein was expressed in *E. coli* in darkness. Purification was performed under minimal illumination and samples were stored in the dark for two weeks afterwards. Subsequent irradiation of cerFP505 with 400 nm light resulted in a full activation of the sample as deduced from the 2% increase of the fluorescence emission (Figure 2B). The emission intensity decreased by ∼15% in response to illumination with 450 nm light but could be fully recovered under 400 nm light irradiation. The intensity differences of the untreated protein, the activated and deactivated states, imply that ∼98% of all chromophores in the ground state equilibrium are found in the active conformation, whereas ∼80% of the photoswitchable fraction adopts a fluorescent state.

After activation, fluorescence emission was recorded in 30 s intervals in the absence of external illumination for 30 min. The intensity did not decrease within this period. Likewise, no increase in emission intensity was recorded for >30 min after deactivation, indicating that the on-switching of cerFP505 is an exclusively light-driven process on the observed minute-time scale (Figure 2C). A bi-stability of the on/off-states for more than half an hour as shown by cerFP505 was recently also observed for Dronpa [71]. In contrast, the photoswitchable proteins KFP and rsFastLime reach the ground state equilibrium by thermal relaxation within a few minutes [16], [71]. The photoswitching of cerFP505 was reversible over numerous cycles of alternating 400/450 nm irradiation (Figure 2D). Under the light conditions used in the experiment (450 nm; ∼80 mW cm^−2^), we determined a half-life of 30 s for off switching. An identical value was calculated for rsFastLime, the fast-switching derivative of Dronpa under similar blue light intensities (∼300 mW cm^−2^) [71]. However, within the time required to switch off rsFastLime completely, only 15% of all cerFP505 molecules can be converted in the dark state. The absence of measurable fluorescence recovery of cerFP505 by thermal relaxation within the time scale of this experiment (Figure 2C) indicates that the off-switching reaction is saturated. This observation suggests the existence of two different conformations of the protein, one that is stably fluorescent and another one that allows photoswitching. Such a durable coexistence of two spectroscopically distinct states has been noticed only for GFP-like red fluorescent proteins (RFPs) with a mixture of green or red fluorescent chromophores [44], [72]–[74].

Most recently, a collection of photoswitchable/photoconvertible FPs including KFP, Dronpa, Kaede, Dendra and EosFP and their biotechnologically engineered variants have enabled imaging of protein localization with a resolution beyond the diffraction barrier of optical microscopy [22], [24], [25], [75], [76]. Microscopy concepts such as RESOLFT (Reversible Saturable Optical Fluorescence Transitions), Photoactivated Localization Microscopy (PALM) [22], [77], Fluorescence Photoactivation Localization Microscopy (FPALM) [78], Stochastic Optical Reconstruction Microscopy (STORM) [79], [80], or PALM with Independently Running Acquisition (PALMIRA) [25] offer great application potential for photoswitchable marker proteins with novel properties. Interestingly, cerFP505 combines the bistability of the on/off-states of Dronpa with the increased switching speed of rsFastLime [71]. These properties allow a tightly controlled switching using comparably low irradiation energy, and are beneficial for local optical marking and super-resolution microscopy. Therefore, cerFP505 appears as important lead structure for the development of a variant in which the stable green fluorescent conformation is converted to the photoswitchable state. Moreover, the discovery of photoswitchable cerFP505 from a deep sea animal reveals the lightless depths of the oceans as a new reservoir of GFP-like proteins with novel and highly desirable properties for imaging applications.

### Eukaryotic expression and imaging of cerFP505

**Figure 3:**
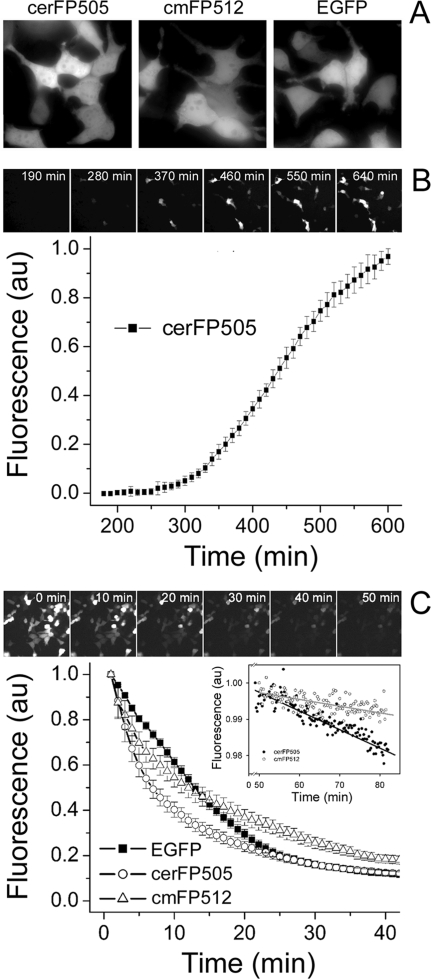
Expression of cerFP505 in mammalian cells. A) cerFP505, cmFP512 and EGFP were expressed in HEK293 cells. Photographs were taken 12 h after transfection. B) The upper panels show the expression and maturation of cerFP505 *in vivo* in HEK293 cells. Images were excised from a time-lapse movie. The time-points of image acquisition are indicated in the panels as minutes after transfection. The increase in fluorescence intensity of cells in the green channel was analyzed for each image of the time-lapse movie. The graph displays the mean of the individual measurement normalized to 1.0, error bars denote standard deviations. C) Photobleaching of cerFP505, cmFP512 and EGFP in HEK293 cells under continuous irradiation with blue light under the fluorescence microscope. The upper panels are images from a time-lapse movie showing the photobleaching of cells expressing cerFP505. Imaging time-points are given and correspond to minutes of irradiation. The graph displays the mean values of the green fluorescence emission of blue-light irradiated cells expressing cerFP505, cmFP512 and EGFP. Error bars denote standard deviations. *Inset*: Photobleaching measured *in vitro* using purified recombinant protein samples.

Deep sea organisms are adapted to a life in constant darkness at temperatures below 10°C. This realization raises the question if their GFP-like pigments would meet the demands on a reporter in mammalian cells, such as functional folding at 37°C and sufficient photostability. Moreover, in cerFP505 as well as in GFP from *Aequorea victoria*, the residue preceding the chromophore is formed by phenylalanine and the exchange of phenylalanine in this position by leucine (Figure 1E) was found necessary to promote functional folding of avGFP at 37°C [9]. The performance of cerFP505 as marker in mammalian cells was tested by transfecting HEK293 cells with an eukaryotic expression vector carrying the cDNA sequence of cerFP505. Transient expression in cells at 37°C showed a uniform distribution without signs of aggregation (Figure 3A). The intensity of green fluorescence was comparable to cells expressing cmFP512 or EGFP under the same conditions. This observation was particularly surprising considering the low-temperature adaptation of the animal that yielded cerFP505. Moreover, eqFP611 from a tropical sea anemone required extensive engineering to create a variant folding efficiently at 37°C [32], [44]. These findings show that the folding of fluorescent proteins is not necessarily correlated with the temperature adaptations of the animals they originated from. It is, furthermore, obvious that a phenylalanine in front of the chromophore does not always impair folding of GFPs at higher temperatures. The fluorescence of cerFP505 in mammalian cells could be already detected ∼6 h after transfection (Figure 3B). The same time period is required by EGFP and cmFP512, indicating that cerFP505 has comparable folding and maturation rates, making the protein suitable for most cell biology applications [43].

Under constant irradiation with blue light (480/40 nm; 1.2 W cm^−2^) under the microscope, cerFP505 displayed faster photobleaching in cells compared to EGFP and cmFP512 (Figure 3C, Table 1). The difference in photobleaching rates of cerFP505 and cmFP512 could also be detected in purified protein solutions at low energy irradiation (450/40; 80 mW cm^−2^) (Figure 3C *Inset*). However, the bleaching rate of cerFP505 is still within the range of values determined for FPs commonly used in imaging applications [27]. Taken together, these results evidence the suitability of GFP-like proteins from deep sea organisms as fluorescent marker for live cell imaging.

### Concluding remarks

We discovered fluorescent deep sea organisms as a new source of GFP-like proteins with highly desirable properties such as reversible photoswitching. In spite of its origin in an animal adapted to conditions hostile for mammalian cells, cerFP505 isolated from a deep sea ceriantharian performed well as a marker protein in a human cell line at 37°C. Furthermore, the identification of an FP from an animal living in an essentially lightless habitat reinforces the emerging idea that proteins belonging to the GFP-like family can exert functions in anthozoans that are not related to photoprotection or the photosynthetic productivity of algal symbionts.

## Materials and Methods

### Animal collection

Using the *Johnson-Sea-Link* submersible (Harbor Branch), tentacle samples were obtained from a green fluorescent ceriantharian found in the Gulf of Mexico at a depth of 530 m. The tentacles were conserved in RNA*later* (Ambion, Foster City, USA) and stored at 4°C for later analysis.

### cDNA library construction and cloning

Total RNA from the deep sea ceriantharian was extracted from tentacle tissue preserved in RNA*later* (Ambion, Foster City, USA) using RNAqueous® (Ambion, Foster City, USA) according to the instructions of the manufacturer. The cDNA library was constructed as described [18] using the SMART cDNA Library Construction Kit (Clontech, Mountain View, USA) and 5′/3′ RACE was performed using the adapter primers in combination with primers designed against cmFP512 ( _5′_ gcagtgatatcacatataaagacaaagttctgcatgg _3′_ ). After sequencing the amplicons, primers were synthesized that bind to the 5′ and 3′ termini of the open reading frame. The full length coding sequence was amplified by PCR and ligated in pQE32 (Qiagen, Hilden, Germany).

### Spectroscopic characterization

Tentacle fluorescence was photographed with a Leica MZ FL III dissecting microscope (Leica Microsystems, Wetzlar, Germany) equipped with a mercury lamp, a standard epifluorescence filter set and a Spot Insight QE digital camera (Diagnostic Instruments, Sterling Heights, USA), whereas spectra of the tentacles were recorded using a USB2000 spectrometer (Ocean Optics, Dunedin, USA) equipped with a fiber optic probe coupled to the eyepiece of the microscope.

The novel GFP-like protein was expressed in fusion with an N-terminal 6× histidine tag in *E. coli* BL21 cells. The protein was purified from the bacterial culture using TALON metal affinity resin (BD Biosciences, Clontech) and size exclusion chromatography as described [43], [44]. Absorption spectra were measured with a Varian Cary 50 Scan UV-Vis Spectrophotometer (Varian, Palo Alto, USA). Fluorescence and photoswitching properties were determined with a Varian Cary Eclipse fluorescence spectrophotometer (Varian, Palo Alto, USA). The molar extinction coefficient was calculated as described for cmFP512 [43]. The fluorescence quantum yield was determined using purified solutions of cmFP512 as reference samples [43]. Photoswitching was accomplished by irradiating the protein sample with a 150 W Xe lamp (LOT-Oriel, Leatherhead, UK), equipped with band pass filters (Schott, Mainz, Germany) with transmission at 400/40 or 450/40 nm (center/bandwidth). The protein solution in the cuvette experienced an energy density of ∼80 mW cm^−2^.

### Eukaryotic expression and imaging

The cerFP505 cDNA was isolated after digestion of pQE32-cerFP505 with Acc65I and BamHI and ligated into the corresponding sites of pcDNA3.1- (Invitrogen, Carlsbad, CA, USA), resulting in pcDNA3.1-cerFP505. HEK-293 cells (ATCC CRL 1573) were grown at 37°C under 5% CO_2_ in Dulbecco's Modified Eagle Medium (DMEM, Invitrogen, Carlsbad, CA, USA) supplemented with 10% fetal calf serum (FCS, Biochrom, Berlin, Germany). For live cell imaging, cells were plated on chambered cover glasses (Nunc, Rochester, NY, USA) at a density of 80,000 cells per chamber in 2 ml medium. After 16 h incubation at 37°C, cells were transfected with 500 ng of pcDNA3.1-cerFP505, pcDNA3-cmFP512 or pcDNA3-EGFP expression plasmid using the Nanofectine transfection reagent (PAA, Pasching, Austria) according to the manufacturer's instructions. 24 h after transfection the living cells were analysed using a fluorescence microscope (DM IRB, Leica Microsystems, Wetzlar, Germany) equipped with a 100-W Hg light source (HO 103W/2, Osram, Munich, Germany) and a standard FITC filterset. Images were taken with a digital camera (C4742, Hamamatsu Photonics, Hamamatsu City, Japan). To study expression and maturation of cerFP505 within transfected cells, time-lapse movies were recorded. Starting 180 min after transfection, frames were acquired in 10 min intervals for a period of 10 h. The fluorescence intensity of four individual cells was analyzed using the ImageJ software (http://rsb.info.nih.gov/ij/). Photobleaching of cerFP505, cmFP512 and EGFP within living cells was analyzed 24 h after transfection. Cells were continuously irradiated with blue light (Excitation filter: HQ480/40, AHF, Tübingen, Germany) with an energy density of 1.2 W cm^−2^. Images were taken every minute for 2 hours. Fluorescence intensity was analyzed as described above, based on five individual image regions each containing ∼6 cells.
